# EZH2 regulates oncomiR-200c and EMT markers in esophageal squamous cell carcinomas

**DOI:** 10.1038/s41598-022-23253-2

**Published:** 2022-10-31

**Authors:** Fatemeh Nourmohammadi, Mohammad Mahdi Forghanifard, Mohammad Reza Abbaszadegan, Vajiheh Zarrinpour

**Affiliations:** 1 Department of Biology, Damghan Branch, Islamic Azad University, Damghan, Iran; 2 Medical Genetics Research Center, Mashhad University of Medical Sciences, Mashhad, Iran

**Keywords:** Cancer, Cell biology, Genetics, Molecular biology

## Abstract

EZH2, as a histone methyltransferase, has been associated with cancer development and metastasis possibly through the regulation of microRNAs and cellular pathways such as EMT. In this study, the effect of EZH2 expression on miR-200c and important genes of the EMT pathway was investigated in esophageal squamous cell carcinoma (ESCC). Comparative qRT-PCR was used to examine EZH2 expression in ESCC lines (YM-1 and KYSE‐30) following the separately transfected silencing and ectopic expressional EZH2 vectors in ESCC. Subsequently, expression of miR-200c and EMT markers was also assessed using qRT-PCR, western blotting and immunocytochemistry. Underexpression of Mir200c was detected in YM-1 and KYSE-30 cells after EZH2 silencing, while its overexpression was observed after EZH2 induced expression. Following EZH2 silencing, downregulation of mesenchymal markers and upregulation of epithelial markers were detected in the ESCCs. Our results demonstrate that EZH2 regulates the expression of miR-200c and critical EMT genes, implying that overexpression of Zeb2, Fibronectin, N-cadherin, and Vimentin lead to a mesenchymal phenotype and morphology while underexpression of epithelial genes, enhance cell migration after enforced expression of EZH2 in ESCCs. EZH2 gene can be a beneficial treatment marker for patients with esophageal cancer through decrease invasiveness of the disease and efficient response to neoadjuvant therapy.

## Introduction

Esophageal cancer (EC) is the seventh most common cancer and the sixth leading cause of cancer-related mortality worldwide with five-year survival rates of less than 20%^1^. EC presents two major histological types including esophageal adenocarcinoma and esophageal squamous cell carcinoma (ESCC)^2^. ESCC is considered as the main histological type (90% of patients) with poor prognosis in the East and the Middle East of Asia, Africa, South America, and Southern Europe^3–5^. Although extensive studies have been performed on the ESCC tumorigenesis, the cellular and molecular mechanisms of ESCC progression and development are inadequately identified. Metastasis, tumor relapses and drug resistance are three major difficulties in the treatment of the ESCC malignancy which are associated with high morbidity and mortality of the disease^6^.

Presented treatments for ESCC are including esophagectomy, local mucosal resection or ablation therapies, chemotherapy, and radiation therapy which are approved by the National Comprehensive Cancer Network (NCCN). For many clinical consequences, there are no sufficient conclusive results to assist EC treatment. Esophagectomy, as one of the most complicated cancer surgeries, has a 5% in-hospital mortality rate and a recovery period of nearly a year^7^. Due to the poor prognoses, esophageal cancer almost found at an advanced stage when traditional treatment modalities cannot accomplish the therapy^8^. Furthermore, a large proportion of treated patients (60–70%) do not respond well to neoadjuant therapies and experience serious side effects (vomiting, fluid and electrolyte imbalance, stomatitis/mucositis, renal, hearing, and peripheral neuropathy^8–10^. As a consequence of ESCC micro-metastasis at the time of clinical examination and diagnosis, most patients with resectable esophageal cancer have a minimum prospect of cure, And ESCC tumor cells had already migrated to distant organs or tissues after surgery^11^. Determining the most effective approach for ESCC patients continues to be extensively investigated as prognoses are frequently rather dismal with many different treatment strategies^7^.

There are different protein-coding genes and key regulators of cancer gene networks which may be involved in ESCC carcinogenesis. Though numerous oncoproteins have been involved in development of ESCC, an increasing extent of research indicates that other types of biological molecules, specially non-coding RNA (ncRNA), are correspondingly essential^12^. Hence, it is critical to identify novel non-invasive biomarkers that can improve diagnosis, prognosis, diagnosis, and treatment of ESCC.

microRNAs (miRNAs) are small (20–24 nt) non-coding RNAs that are contributed in post-transcriptional regulation of gene expression in multicellular organisms by affecting both the permanency and translation of mRNAs^13^. According to their target genes and the tissues in which they are expressed, miRNAs are classified into different groups such as oncomiRs and tumor suppressors^14^. Specific miRNAs have been found in various cancers having a strong impact on the development of human tumors and the prognosis of patients and abnormal miRNA expression has been identified in a variety of cancers^15^, implying miRNAs as a possible therapeutic target for cancer care^16^. miRNA expression monitoring is important as either a valuable diagnostic or prognostic tool^17^. Moreover, it has been demonstrated that miRNAs are present in human plasma in an extraordinarily stable shape that is protected from endogenous RNase activity^18,19^. MicroRNA (miR)-200 family members are consisting of miR-200a, miR-200b, miR200c, miR-141, and miR-429, which controlled invasion and metastasis in different advanced tumors^20^. miR-200c has key functions in cancer proliferation, metastasis, cell cycle regulation, invasion, and apoptosis in many cancer cells^21^. In addition, miR-200c is a well-known prognostic and diagnostic marker in a variety of cancer types^22^. The oncogenic microRNA, miR-200c has been discovered recently in ESCC^23^.

Since ESCC is known as an aggressive disease, not only most patients are diagnosed at late stages of carcinogenesis, but also tumor migration to distant organs and tissues is detected after surgery. Therefore**,** investigation of probable components involved in this process may help to a better inhibition of ESCC invasiveness and aggressiveness^8,11^. EMT is considered as one of the main promoters of invasion and metastasis in cancer. Through EMT, epithelial cancer cells acquire migration and invasive mesenchymal cell-like features, allowing them to emigrate from the initial tumor mass and spread to distant locations^24^.

The Polycomb proteins (PcG) are important components of histone modifier^25^. They are known as transcriptional suppressors primarily by causing chromatin remodeling and controlling the expression of a variety of developmentally regulated genes^26^. The PcG forms at least two main repressive complexes: Polycomb repressive complex 1 (PRC1) and Polycomb repressive complex 2 (PRC2)^27^. Enhancer of zeste homolog 2 (EZH2) is a component of the polycomb repressive complex 2 (PRC2), which catalyzes the trimethylation of lysine 27 in histone 3 (H3K27me3) it mediates chromatin compaction that induces transcriptional repression of target genes^28,29^. EZH2 controls genes involved in EMT and invasion targeting PRC2 potentially affects tumor metastasis and angiogenesis^30^. EZH2, can regulate key genes involved in the recurrence and progression of cancer^31^. EZH2 promotes and regulates EMT, as well as migration and invasion in several cancers^32^. In a variety of malignant tumor models, EZH2 mediates H3K27me3 and plays a key role in driving tumor growth and metastasis^33–35^. Furthermore, gain or loss of function mutations in EZH2 have been found in different cancers^34–36^. Since epigenetics regulatory pathways and polycomb proteins such as EZH2 are associated with EMT^29^ and tumorigenesis of different malignancies**,** we aimed in this study to explore the functional correlation between EZH2 and Mir200c in ESCCs as well as evaluate the impact of EZH2 on EMT genes (E-cadherin, Occludin, Vimentin, Fibronectin, N-cadherin, Zeb2) main components to reveal a mechanistic route for EZH2 in this process.

## Materials and methods

### Cell lines and culture condition

ESCC lines YM-1 and KYSE30 were cultured in RPMI-1640 and Dulbecco’s modified Eagle’s medium/-F12 media medium (PAA, Pasching, Austria, and Gibco, Grand Island, NY), respectively, supplemented with 100 U/ml streptomycin/penicillin (Gibco) and 10% heat-inactivated fetal bovine serum (FBS, Invitrogen, Carlsbad, CA) at 37 °C in a humidified atmosphere containing 5% CO2. KYSE-30 cell line was purchased from the Pasteur Institute Cell Bank of Iran (http://ncbi.Pasteur.ac.ir/) and YM1 cells were purchased from Department of Molecular Medicine, Faculty of Advanced Medical Technologies, Golestan University of Medical Sciences, Gorgan, Iran.

### EZH2 gene expression study

**Figure 1:**
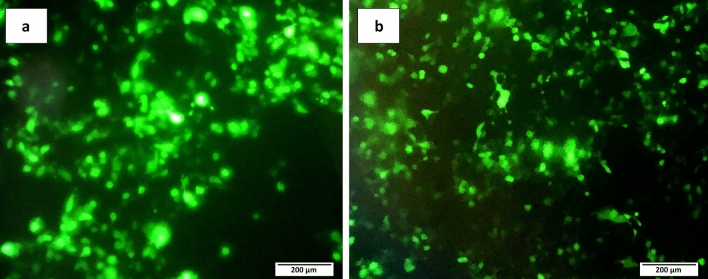
Invert and fluorescent microscope images of GFP transfection efficiency of GFP gene expression in (**a**) YM1 and (**b**) KYSE-30 cells. Fluorescent microscopy images of GFP control cells 24 h after transfection.

EZH2-specific short hairpin RNA (shRNA) (RNAi-Ready pSIREN-RetroQ Vector, kindly provided by Dr. Moein Farshchian, (Molecular Medicine Research Department, ACECR-Khorasan Razavi Branch, Mashhad, Iran) was used to downregulate EZH2 in YM-1 and KYSE-30 cells^37^. In addition, the Human EZH2 ORF mammalian expression plasmid, C-HA tag (Sino Biological Inc. Catalog Number: HG11337-CY) was used to upregulate EZH2 in the cells. Green fluorescent DNA plasmid (GFP) expression vector pCDH-513b (System Bioscience, Palo Alto, CA) was also used as a control plasmid for transfection efficiency. Cells were transfected using X-treme Gene HP DNA transfection reagent (Roche, Basel, Switzerland). Briefly, approximately 600,000 cells were seeded per six-well plate 18 h before transfection. The growth medium was exchanged with serum/antibiotic-free RPMI-1640 and DMEM medium 3 h before transfection. The cells were transfected with a total of 1 μg DNA per well. 6 h post-transfection the cells were supplemented with FBS, and incubated for 48 h. After separately transfection of EZH2 silencing and inducing vectors, transfection efficiency was checked in YM-1 and KYSE-30 GFP‐control cells (Fig. 1). Consequently; cells were treated with trypsin and EDTA (brand trypsin and EDTA CegrogenN0100-751) 48 h after transfection and subjected to RNA extraction.

### RNA extraction, cDNA synthesis, and qRT-PCR

The total RNA, including mRNA and miRNA, was isolated from 1 × 10^6^ of both EZH2-silenced and EZH2-overexpressed KYSE-30 and YM1 as well as the control cells using the total RNA extraction kit (Pars Tous, Iran), according to the manufacturer’s instruction. Purity and amount of total RNA were measured on a Nano Drop spectrophotometer (WPA, Biowave II, and Germany). In addition, RNA integrity was assessed by 1% agarose gel electrophoresis, and 28S and 18S ribosomal RNA bands were observed accordingly. Afterward, the DNA decontamination steps were performed using Thermo Fisher Scientific kit (USA) according to the manufacturer’s protocol.

Thermo fisher kit (Catalog number: K1621) and micro Script microRNA cDNA synthesis kit (Norgen Cat. 54410) were used for cDNA synthesis based on the manufacturer’s instruction.

The expression of EZH2, mir200-c, E-cadherin, Occludin, Vimentin, Fibronectin, N-cadherin and Zeb2, was evaluated in EZH2-silenced and EZH2-overexpressed cells compared to control using relative comparative real-time PCR analysis. Comparative real-time PCR (SYBR^®^ Premix Ex Taq II Kit, TaKaRa) was performed in duplicate reactions (Light Cycler, Roche, Germany) with specific primers (Table 1) to determine EZH2, miR-200c, E-cadherin and Zeb2 mRNA expression levels. Data were normalized by glyceraldehyde-3-phosphate dehydrogenase (GAPDH) for EZH2, EMT genes and Zeb2, while U6snRNA was used as normalizer for miR-200c. The thermal profile for EZH2 and EMT genes was consisted of an initial denaturation step at 95 °C for 2 min, 45 cycles (95 °C for 30 s and 60 °C for 30 s) for EZH2 and (95 °C for 30 s and 61 °C for 30 s) for E-cadherin, Occludin, Vimentin, Fibronectin, N-cadherin and Zeb2, a final extension step of 72 °C for 30 s. The used thermal profile for miR-200c was a first denaturation step of 95 °C for 3 min, after that 40 cycles of (94 °C for 30 s and 60–63 °C for 30 s) and an ending extension step of 72 °C for 45 s. The 2 − ΔΔCT method was used to analyze the expression level of the genes. (Table.1).Tumors with more and less than 2 and − 2 log2 fold change was considered as overexpressed and under expressed, respectively. The log2 fold change between 2 and − 2 was defined as normal or unchanged expression.

**Table 1:** Primer sets sequences used in quantitative real-time PCR.

Gene	Primers sequence
mir-200c-F	TAATACTGCCGGGTAATGATGGA
U6 RNA-F	GTGCTCGCTTCGGCAGCACATAT
Universal R	GAATCGAGCACCAGTTACGC
E-cadherin F	ATTCTGATTCTGCTGCTCTTG
E-cadherin R	AGTCCTGGTCCTCTTCTCC
Zeb2 F	GGGACAGATCAGCACCAAAT
Zeb2 R	CGCAGGTGTTCTTTCAGATG
Vimentin F	GGCTCGTCACCTTCGTGAAT
Vimentin R	GAGAAATCCTGCTCTCCTCGC
GAPDH F	GCCATCACGCCACAGTTTCC
GAPDH R	TTCGGGAACCAAGCTACACC
Fibronectin F	AGGAAGCCGAGGTTTTAACTG
Fibronectin R	AGGACGCTCATAAGTGTCACC
Occludin F	AAGCAAGTGAAGGGATCTGC
Occludin R	GGGGTTATGGTCCAAAGTCA
N-cadherin F	ATGGTGTATGCCGTGAGAAG
N-cadherin R	TGTGCTTACTGAATTGTCTTGG

### Cell migration assays

Cell migration was examined using wound healing assay. For this reason, after transfection, cells were plated in 6-well and allowed to forming a monolayer, and then the cell surface was, wounded by a p-200 pipette tip. The wound closure was monitored at baseline, 12 and 24 h later, using an inverted microscope (Nikon, Tokyo, Japan). Image J 7.0 was used to analyze the minimum distance of wound borders, and all tests were executed in triplicate.

### Immunocytochemistry (ICC)

YM-1 and KYSE-30 cell lines were transfected as described above and collected in Immunocytochemistry fixing solution (FS) (4% paraformaldehyde and 0.05% v/v F68). Cells smear was prepared by celltrazone huro (path filter-Korea). Concisely, following peroxidase blocking specific monoclonal antibody incubation was started for 30 min at 25 centigrade degree. The used antibodies include Cadherin-E master diagnostics (Mouse anti-human Cadherin E Monoclonal Antibody/Clone HECD-1) and. Anti-beta Actin (antibody (ab8227) Abcam). After rinsing for removing excess antibody, secondary antibodies (Sigma, RE7111, US) was applied. Immunocytochemical identification was performed by the Novolink™ DAB (Polymer) (Product No: RE7230-K, RE7270-K) chromogen according to the manufactures’ protocol. Lastly, hematoxylin was applied as a counterstained. All photos were captured via a digital camera (Olympus image analysis system, Japan). Two expert pathologists who were blinded to the primary research, independently scored the immunocytochemical staining of, E-cadherin, according to the semi-quantitative immunoreactivity score. The intensity of immunostaining was scored as 0–3 (0, negative; 1, weak; 2, moderate; and 3, strong), and the percentage of immunoreactive cells was analyzed utilizing Image J software.

### Western blot analysis

Thermo Fisher’s Pierce RIPA Buffer was used to lyse the cells, along with a phosphatase and protease inhibitors cocktail (Roche-04906845001, Sigma-Aldrich). The cell lysates were firstly separated on SDS-PAGE gels, transferred to polyvinylidene fluoride (PVDF) membranes from Millipore, and then blocked with 5% milk for 1 h at room temperature. After washing, PVDF membranes were treated with the primary antibody (Cadherin E master diagnostics (Mouse anti-human Cadherin E Monoclonal Antibody/Clone HECD-1) and Anti-beta Actin antibody (ab8227) Abcam) for one hour at room temperature and then incubated with the secondary antibody overnight at 4 °C. Luminescence substrate was used for visualizing band and G-Box gel documentation system (Syngene, Cambridge, UK) detected and captured images. Densitometry quantification was carried out via ImageJ software.

### In silico analysis

GeneMANIA web base database was used to predict potential targets of EZH2. For the has-miR-200c board, MiRWalk3.0 (http://mirwalk.umm.uni-heidelberg.de/) was used to predict has-miR-200c target genes. The miRWalk platform is based on projected mRNA targets and combines predictions from several prediction tools, including miRDB, TransmiR, target scan Human, and miRTarbase. Also, RPISeq website was used to predict interaction between miR-200c and EZH2.

### Statistical analyses

The statistical analyses were performed using the Graf Pad PRISM 8 statistics software (Graph Pad Software, San Diego, CA, US). P value < 0.05 was regarded as statistically significant. Sample t-test and Pearson association tests were applied to determine the correlation between the genes.

### Ethical approval and consent to participate

The study was approved by the Damghan branch, Islamic Azad University ethical guidelines.

## Results

### EZH2 correlates with miR-200c and EMT key genes in ESCC

**Figure 2:**
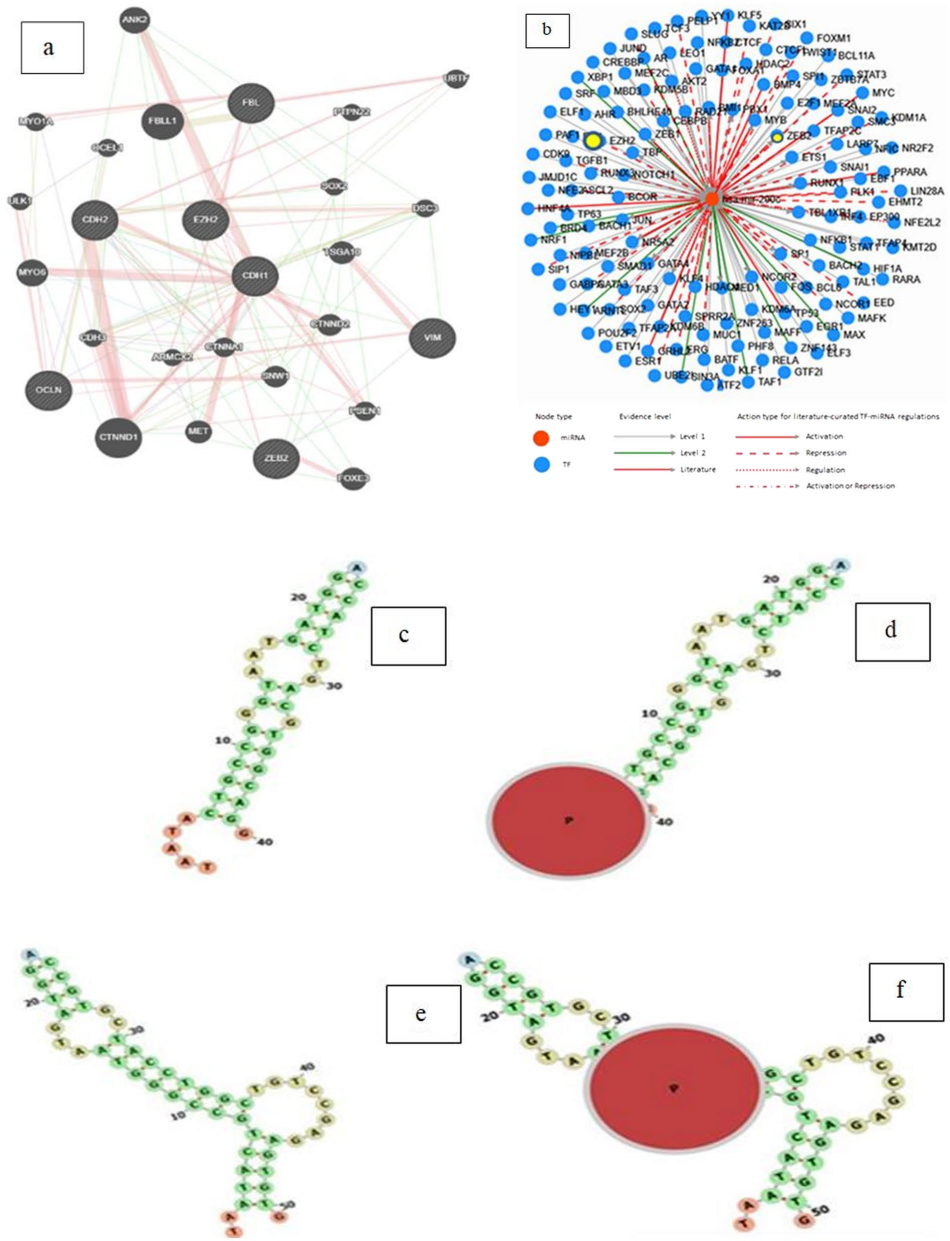
(**a**) The predicted protein–protein interaction network based on GeneMANIA. Predicted: predicted EZH2 relationships via EMT genes. (**b**) The image of interacted transcription factors with miR-200c which predicted by TransmiR database (http://www.cuilab.cn/transmir) network. (**c**) 3D image of has-miR200c-3p A form. (**d**) 3D image of has-miR200c-3p and interaction between EZH2 protein and TAATA 5ʹ end of has-miR200c-3p. (**e**) 3D image of has-miR200c-3p B form. (**f**) 3D image of has-miR200c-3p and interaction between EZH2 protein and (CCGGGTA) of has-miR200c-3p.

In order to determine EZH2 interaction with miR-200c and EMT genes we recruited bioinformatics tools. Based on GeneMANIA web-based bioinformatics resource prediction, EMT genes interact with EZH2. GeneMANIA predicted a direct association between EZH2 and E-cadherin, and Zeb2 (Fig. 2a). For investigating EZH2 and has-miR-200c correlation TransmiR was utilized. The results predicted that has-miR-200c interacts with 136 transcription regulators, including EZH2 and Zeb2. According to TransmiR has-mir-200c interacts with EZH2 with 3.3835 Fold, 0.2959 P-value, and 0.4968 FDR (Fig. 2b). Web-based bioinformatics target scan Human showed that the E-cadherin, Fibronectin, and Zeb2 genes may be targets for has-miR-200c 3p and 5p. Furthermore, interaction between EZH2 and hsa-miR-200c was predicted by using miRWALK and RPISeq websites. We observed a significant score prediction in miRWALK (0.84) that demonstrated has-miR-200c3P may bind to the coding sequence (CDS) of EZH2 mRNA, consists of the score 0.92 binding sites 18,691,886 nt positions in the CDS of EZH2. In 2D form with Energy − 20.9 G and Bind Length of 17 nucleotide to sha-mir-200c. Having used PDB and Frona (http://rna.tbi.univie.ac.at/forna) tools we found that EZH2 has an affinity to bind TAATA 5ʹ end of has-miR-200c in A form. (Fig. 2c,d) Another 2D form revealed that mir-200c3P may bind to the CDS site of the EZH2 gene with Energy − 18.9 G and Binding Length of 28 nucleotides including 9 to 15 nucleotide B form of has-miR-200c CCGGGTA (Fig. 2e,f). We also found has-miR-200c can bind to EZH2 gene according to RF classifier with 0.6 scores and SVM classifier with 0.91 score in RPISeq website. (http://pridb.gdcb.iastate.edu/RPISeq/results.php), it predicts interaction probabilities ranging from 0 to 1. Predictions with probability > 0.5 were considered “positive” in performance evaluation experiments, indicating that the related has-miR-200c and EZH2 are likely to interact. In cross-validation evaluation studies on data sets, classifier accuracies range from 87 to 90%.

### EZH2 regulates miR-200c expression in ESCC

**Figure 3:**
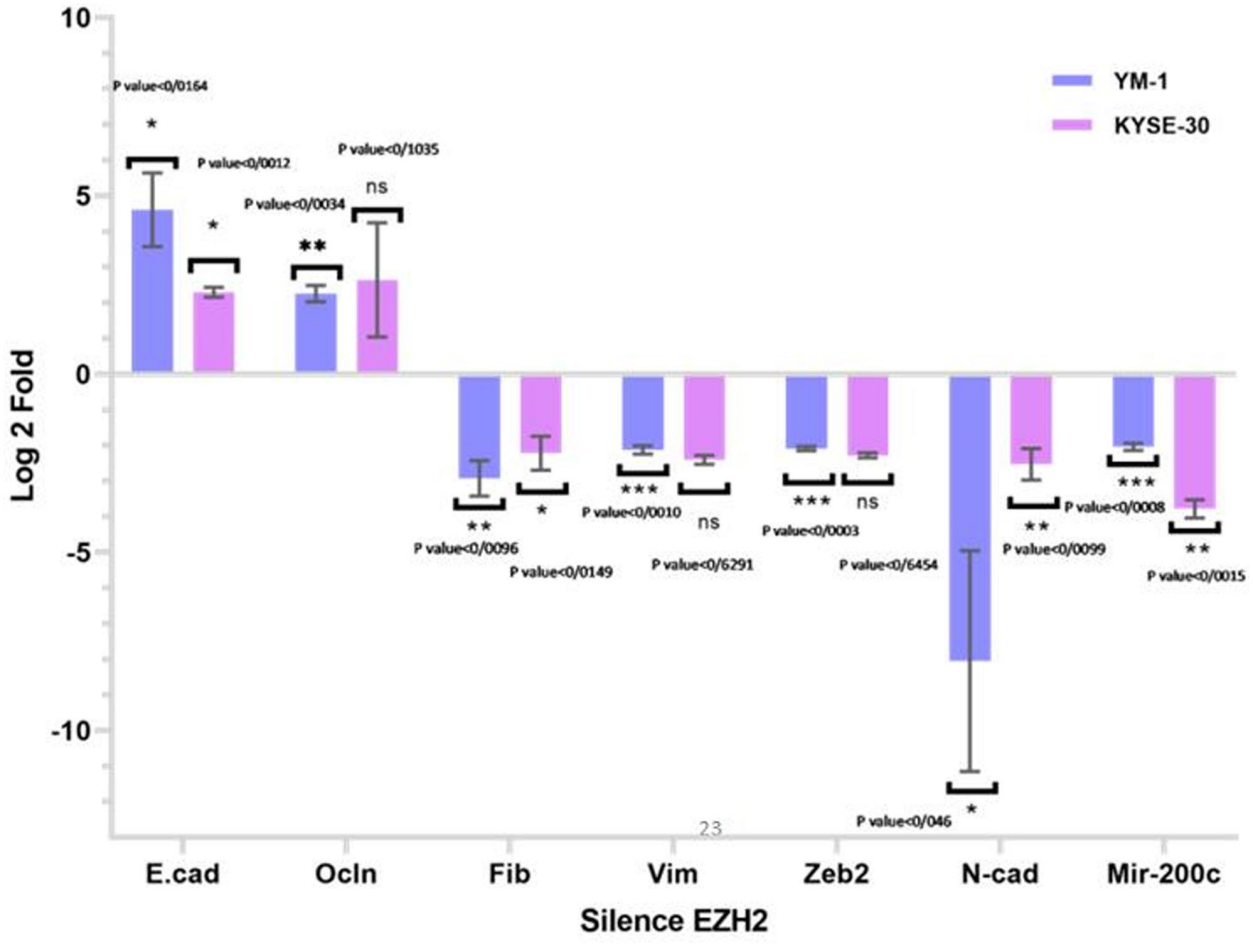
EZH2 silencing led to downregulation of miR-200c and mesenchymal genes, and upregulation of epithelial genes expression in ESCC lines. miR-200c expression decreased nearly − 2.04 log2 fold change in YM-1 cells and − 3.78 in KYSE-30 cells, moreover it caused a decrease in mRNA level of ZEB2, Fibronectin, N-cadherin and Vimentin in ESCC lines while the epithelial genes E-Cadherin and Occludin were upregulated.

**Figure 4:**
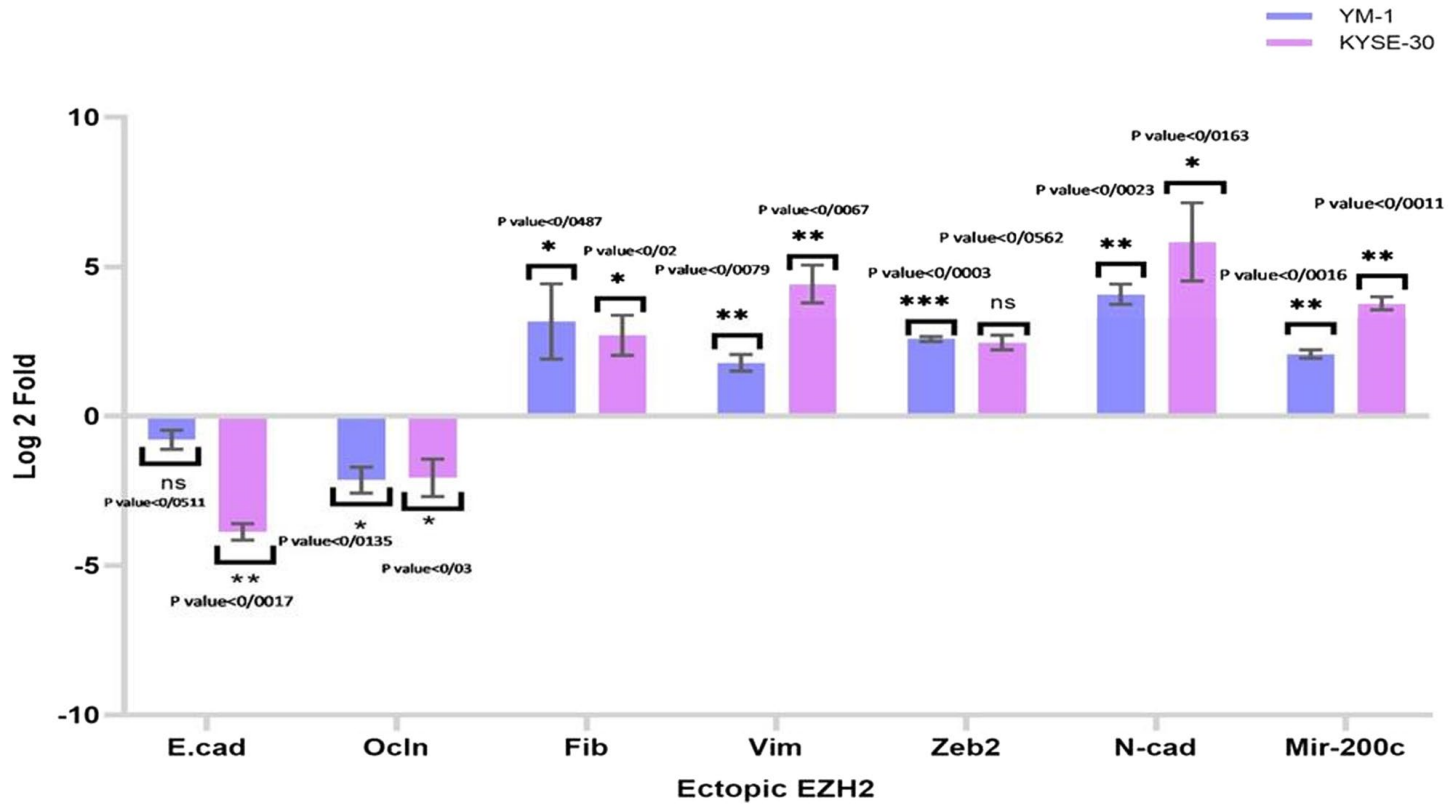
In our study demonstrated that Eectopic expression of EZH2 in ESCC lines upregulates Mir-200c and mesenchymal genes expression while down-regulates expression of epithelial gene. EZH2-overexpressing cell lines YM-1 and KYSE-30 overexpressed miR-200c nearly 2.07 and 3.81-folds compared to control cells, as well as an increase in ZEB2, Fibronectin, N-cadherin and Vimentin mRNA levels, and a decrease in the level of the epithelial gene expression (E-Cadherin and Occludin). The use of an asterisk (*) denotes statistical significance.

Following silencing of EZH2 mRNA in YM-1 and KYSE-30 cells, quantitative real-time PCR (qRT-PCR) was used and we found that miR-200C level was repressed in both EZH2 silenced ESCC lines (Fig. 3). The results showed that has miR-200c may be regulated by EZH2 in the cells. Real-time PCR verification was performed to verify the miR-200C level after EZH2 enforced expression in the ESCC cell lines. We found that in EZH2 induced cells, has-miR-200c was also overexpressed compared to controls cells (Fig. 4). These findings clearly revealed that EZH2 has a significant impact on miR-200c expression in ESCCs.

### EZH2 modulated the EMT components in ESCC

Tumor metastasis and cell migration modulated by the epithelial mesenchymal transition (EMT) pathway are important in the development of various cancers^38^. Since many aggressive tumors were reported to overexpress EZH2, it was suggested that this protein act as an important regulator of the development of EMT^39^. Consistent with the previous prediction with web-based bioinformatics, we examined the mRNA level of the EMT genes in EZH2-treated YM-1 and KYSE-30 cells. While EZH2 silencing caused a significant decreased expression of Vimentin, Fibronectin, N-cadherin, and Zeb2, it caused a meaningful increase in expression of E-cadherin and Occludin (Fig. 3, Table 2). In EZH2-overexpressed cells we identified downregulation of E-cadherin and Occludin expression and upregulation of Vimentin, Fibronectin, N-cadherin and Zeb2 in YM-1 and KYSE-30 cells (Fig. 4, Table 2).

**Table 2:** Log2 fold change of genes assessed via quantitative real-time PCR.

Gene	EZH2-Silenced KYSE-30	EZH2-Silenced YM-1	EZH2-Ectopic KYSE-30	EZH2-Ectopic YM-1
Vimentin	− 2.09	− 2.04	4.42	1.78
Fibronectin	− 2.32	− 2.92	2.7	2.99
Zeb2	− 2.29	− 2.13	2.58	2.46
Occludin	2.64	2.25	− 2.05	− 2.13
N-cadherin	− 2.53	− 8.05	5.83	4.07
E-cadherin	2.29	4.6	− 3.86	− 0.78
Has-miR-200c	− 3.78	− 2.04	3.81	2.07
EZH2	− 4.7	− 9.7	3.86	9.53

### EZH2 accommodate cell migration in ESCC

**Figure 5:**
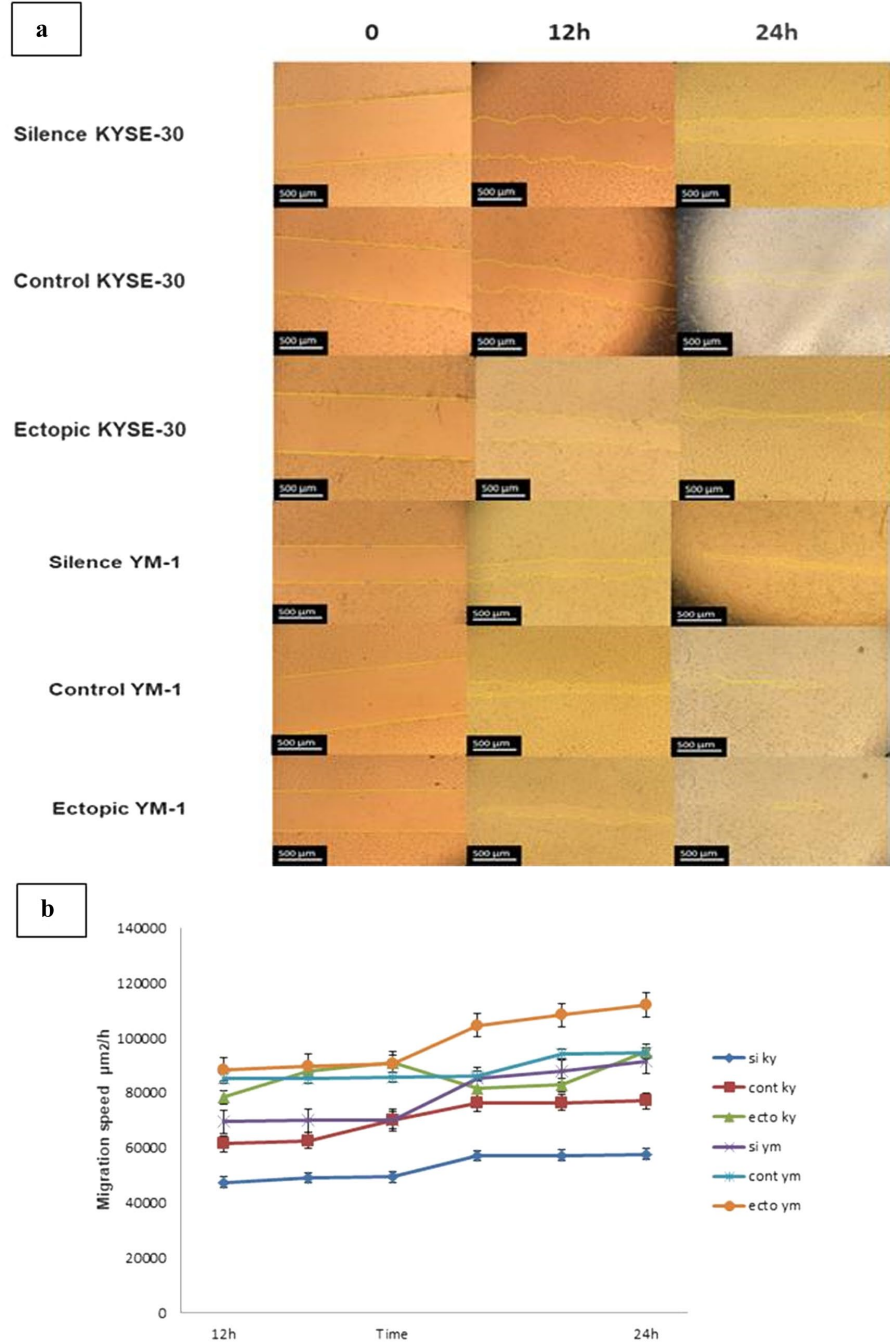
(**a**) Wound healing assays in ESCC lines treated overexpressing and underexperssion of EZH2 relative quantification of the effects observed anlyzed by Image j software. (**b**) Migration speed of ESCC cells was demonstrate more migration speed in EZH2-ectopic cells (μm^2^/h).

**Figure 6:**
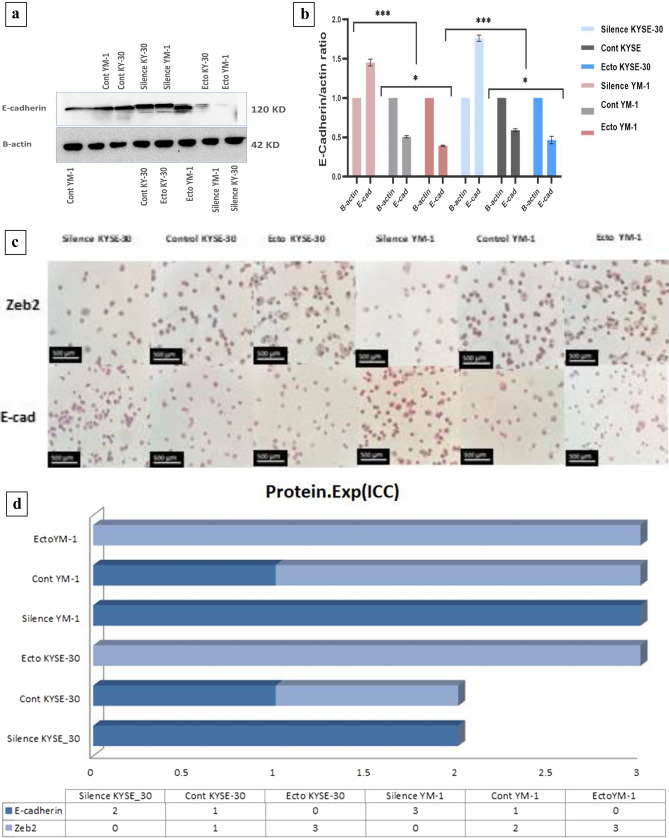
(**a**) WB Image analysis with the G-Box gel documentation system. (**b**) WB analysis confirmed high level of E-cad protein experssion in EZH2-ectopic cells. B-actin antibodies was used as a control. (**c**) Positive staining corresponds to Iimmunocytochemistry staining of E-cad and Zeb2 protein (dark brown is strong positive). (**d**) patologist score to E-cad and Zeb2 protein expersion via Immunocytochemistry assay (3 is srongly positive) (Supplementary Information).

**Figure 7:**
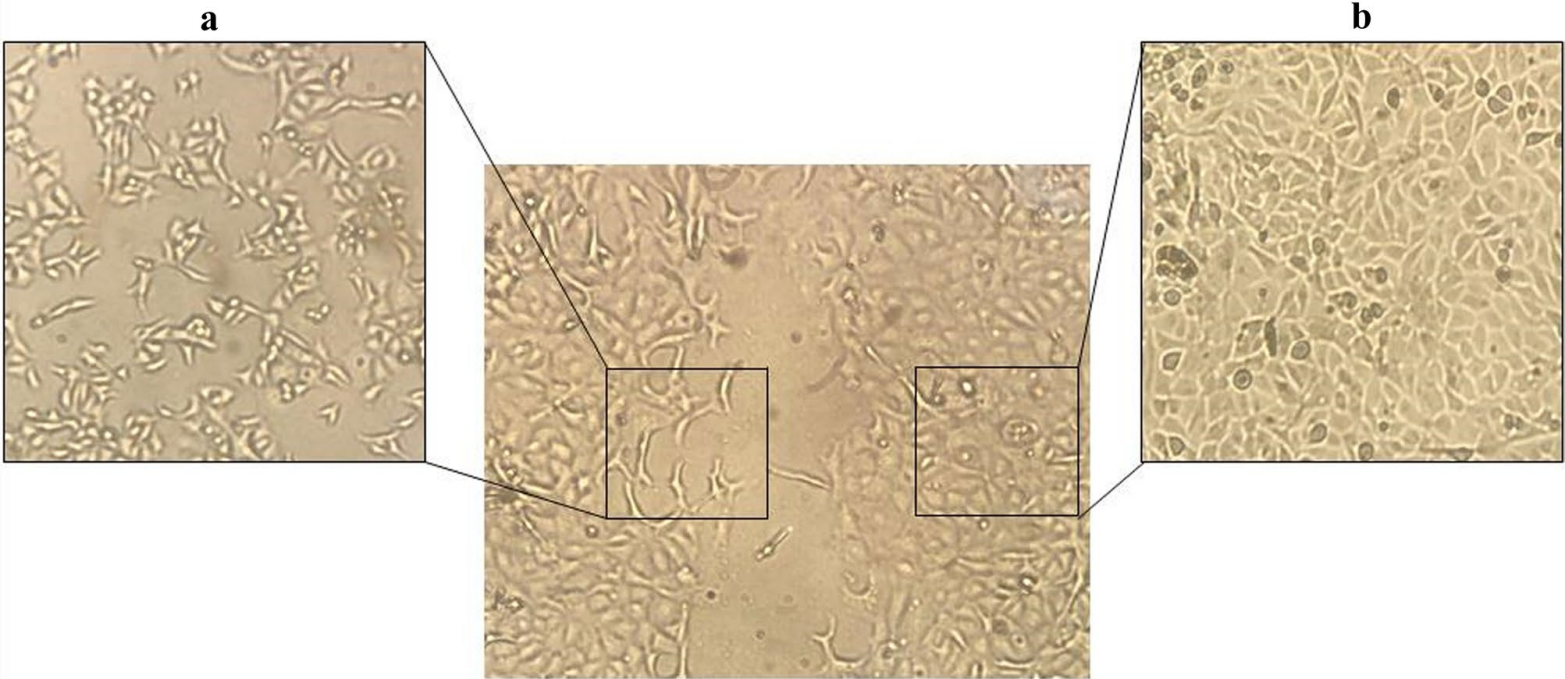
Invert microscopy identifies morphological changes induced in ESCC lines by EZH2 at 48 h. (**a**) Epithelial morphology. (**b**) Mesenchymal morphology.

To substantiate the results of q-RT-PCR and protein expression assays, in vitro migration was assessed through wound healing assay in esophageal cancer cells. After scratching, the area of gap was measured through 24–72 h. The results revealed that EZH2-overexpressed YM-1 cells close the scratched gap approximately three times faster than control cells. Besides, wound healing assay experiments demonstrated a significant decrease in migration of both EZH2-silenced YM-1 and KYSE-30 cells in comparison with the controls, which demonstrated the impact of EZH2 on migration process in ESCCs (Fig. 5). We alternatively examined E-cadherin and Zeb2 in protein level confirming the q-RT-PCR results. As expected, immunocytochemistry staining and western blotting outcome indicated E-cadherin repression in EZH2-overexpressed cell lines while its enhanced expression in EZH2-silenced cells (Fig. 6). The epithelial-to-mesenchymal transition is regarded as one of the crucial constituents of metastatic progression in vitro. The epithelial phenotype is distinguished by a rounded morphology and a spindle-shaped morphology modified to facilitate invasion and migration. EZH2 can control the plasticity of the transition between epithelial and mesenchymal escapes, which is increasingly recognized as a dynamic process in the development of cancer^40^. EMT induction through EZH2 overexpression in ESCC was demonstrated by changes in cellular morphology from epithelial to mesenchymal which led to metastasis and invasion in EC (Fig. 7), a reduction in E-cadherin protein levels likewise Occludin at mRNA level, and an increase in Vimentin, Fibronectin, N-cadherin and Zeb2 expression levels (Fig. 4). The morphological modifications started to be noticeable between 12 and 24 h after EZH2 induction and progressed during EZH2 transfection up to 48 h (Fig. 7).

### Inhibitory effect of EZH2 on E-cadherin gene expression in ESCC

E-cadherin is a key homophilic cell–cell adhesion protein that prevents the migration of individual cells on a matrix. In metastatic cells E-cadherin expression is down-regulated^41^. To further demonstrate that EZH2 mediates migration of ESCC, we performed western blot and immunocytochemistry to assess protein level of E-cadherin and Zeb-2. According to western blotting, E-cadherin was notably upregulated in the EZH2-silenced cells, while it was downregulated in EZH2-overexpressed cells (Fig. 4E). Furthermore, immunocytochemistry staining revealed E-cadherin overexpression and Zeb2 underexpression in EZH2-silenced cells. These findings suggest that silencing EZH2 suppressed EMT and metastatic progression in ESCC by regulating E-cadherin and other EMT key genes.

## Discussion

**Figure 8:**
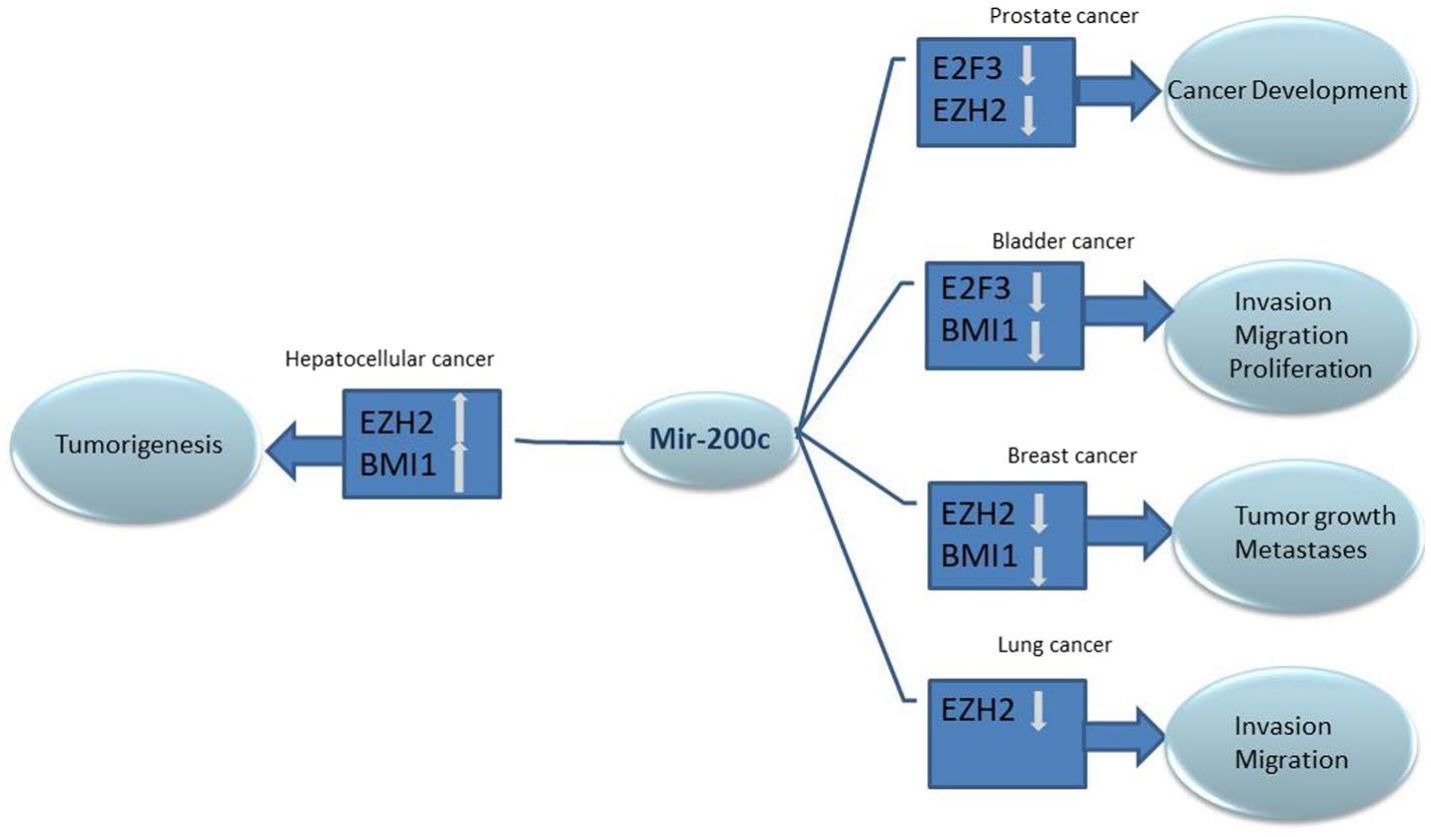
An overview of the role of mir-200c in various cancers: E2F3 controls EZH2 expression at the transcriptional level in prostate cancer, MiR-200c can regulate E2F3 translation directly by targeting its mRNA 3′ UTR and cause a decrease in E2F3 level in the cell leading to a significant decline in EZH2 gene expression^52^. In line with this evidence, an association was detected between miR-200c overexpression and down regulation of E2F3 and BMI-1 genes in bladder cancer^53^. miR-200c plays a tumor suppressor role in hepatocarcinoma, EZH2 can epigenetically silence miR-200c leading up-regulation of BMI-1 followed by an increase in proliferation and metastasis of the malignancy^55^. Interestingly, the expression of the stem cell-associated genes EZH2 and Bmi1 was significantly reduced in breast cancer cells after miR-200c induction metastasis and tumor growth^56^. miR-200c is increased in prostate, breast, bladder, and lung cancers. miR-200c reduces EZH2 and E2F3 genes that cause tumor development in prostate cancer. In bladder cancer, reducing E2F3 and BMI1 genes reasons metastasis and cancer proliferation. In breast cancer downregulating EZH2 and BMI1 genes, affects tumor growth and metastasis. In contrast, in hepatocellular cancer miR-200c knockdown increased EZH2 and BMI1 leading to tumorigenesis.

Esophageal cancer is one of the most prevalent malignancis in both gender men and women. The most prevalent histological type is ESCC, identified as tumor present with extensive metastases^42^. The tumor initiation, development, metastasis, and tumor recurrence in esophageal cancer, significantly influenced through EMT pathway^43^. In addition, MiRNAs have been considered to regulate cell proliferation, apoptosis, epithelial-mesenchymal transition (EMT), metastasis, chemotherapy and radiotherapy through playing an oncogenic role^44^. Considering metastasis as the main cause of more than 90% of cancer-related mortalities, elucidating the molecular processes by which microRNAs promote metastasis and identifying new therapeutic targets is critical^45^ miR-200c plays the role of an oncomir in ESCC, and many malignant cancers (thyroid carcinoma, testicular germ cell tumors, rectum adenocarcinoma, pancreatic adenocarcinoma, kidney renal papillary cell carcinoma, and cervical squamous cell carcinoma)^20,44,46–50^. Moreover, miR-200c regulated EZH2 through BMI-1 and E2F in breast, lung, prostate, bladder cancers and hepatocarcinoma. MiR-200c was regulated by EZH2 through epigenetic repression in prostate cancer, and miR-200c directly controlled the expression of E2F3 by targeting 3′UTR. E2F3 positively conducted the transcriptional level of EZH2^51^ (Fig. 8). In gastric cancer SNHG22 LncRNA recruits EZH2 and sponge mir-200c-3p to suppress tumor suppressor genes and promote the cancer progression^52^. In lung cancer cells, miR-200c overexpression significantly prevented cell migration and invasion by increasing the level of E-cadherin and decreasing the expression of EZH2^53^.

MiR-200c is overexpressed in esophageal cancer and promotes chemotherapy and radiotherapy resistance in tumor cells. ESCC patients with high-serum level miR-200c may have a higher risk of death than someone with low expression^54^. Hence, serum miR-200c levels can be used to predict chemotherapy responses and prognosis in patients with esophageal cancer^54^. Previous studies reported the correlation between EZH2 and miR-200 family in a few cancer types including hepatocellular, prostate, and OSCC malignancies (Fig. 8). Although, the effect of the miR-200 expression on EZH2 has been examined, no study investigated inversely describing that how EZH2 can regulate miR-200 family. In this study, we have evaluated the effect of the EZH2 gene on miR-200c and EMT genes in esophageal squamous cell carcinoma.

EZH2 can directly regulate EMT by inhibiting E-cadherin and Occludin expression^55,56^. EZH2 is one of the most important members of polycomb family which is upregulated in ESCC and promotes tumor development. EZH2 is found in a variety of cancers, including breast, prostate, bladder, colon, lung, and pancreatic cancers, as well as anaplastic thyroid, nasopharyngeal, endometrial carcinomas, and lymphomas^29,35,56^. Overexpression of EZH2 is strongly associated with advanced stages of cancer progression, and tumor metastasis by regulating EMT pathway in a variety of cancers^29,39,57^. For instance, EZH2 knockdown upregulated the expression of E-cadherin in ovarian cancer cells^58^. Likewise, in lung cancer cells with enhancing EZH2 protein expression, EMT was regulated through upregulation of Vimentin and reducing of E-cadherin^59^. EZH2 can directly associate with EMT in downregulating E-cadherin expression by histone H3K27 trimethylation and indirectly through miR-361 suppressing endometriosis. Moreover, EZH2 enhances expression of mesenchymal markers (Vimentin, N-cadherin, and Fibronectin) leading the promotion of EMT pathway in endometriosis^55^. In gastric cancer cells EZH2 promotes EMT, upregulates Vimentin and downregulates E-cadherin. By direct binding to the PTEN locus, EZH2 reduces PTEN expression subsequently triggers the Akt pathway, and ultimately leads to the acquisition of metastasis and pluripotent phenotype^60^. Different EZH2-mediated gene promoter methylation have been demonstrated to increase the malignant phenotypes of cancer cells, which is related to the loss of tumor suppressor gene activities and results in stem cells or progenitor cells developing to cancer cells^52^.

Our results demonstrated a close correlation between EZH2 expression and epithelial-mesenchymal markers expression, supporting a critical aspect of EZH2 in cellular EMT process.

Following EZH2 downregulation in ESCCs, the level of miR-200c and mesenchymal genes dropped significantly and epithelial markers (E-cadherin, and Occludin) expression was promoted, while after ectopic expression of EZH2 in esophageal squamous carcinoma cell lines, expression of miR-200c and mesenchymal genes (Vimentin, Fibronectin, N-cadherin and Zeb2) were increased and epithelial markers expression were suppressed compared to control cells. EZH2 is recognized to play an important role in these two crucial aspects of cancer biology. Downregulating EZH2 may prevent the progression of ESCC.

To the best of our knowledge, the current study was the first to evaluate the effect of EZH2 gene expression on miR-200 in ESCC and show a connection between EZH2/miR-200c and prognosis in ESCC. The molecular mechanism between miR-200c and EZH2 is complicated and further research is necessary to clarify the role of the EZH2/miR-200c and probable involved proteins in ESCC carcinogenesis.

The findings in this study may help therapeutic approaches in two ways. First, reducing the expression of the EZH2 gene, which reduces cancer cell progression, invasion and metastasis trough EMT pathway, and second, by reducing miR-200c expression, which increases patients’ survival and response to chemo and radiotherapy.

In conclusion, EZH2 can be an effective therapeutic marker for esophageal cancer patients. Our results demonstrate that by repressing the expression of EZH2 in esophageal cancer cells, the EMT pathway and as a result, cell migration and metastasis are reduced, which can increase the hopefulness of esophageal cancer patients for treatment without recurrence and increase the survival rate in patients. This study provided an understanding of the critical activities of the EZH2, which may be affected by miR-200, in ESCCs, although its regulating cellular mechanisms are still ambiguous and required further investigation.

## Supplementary Information

Supplementary Information.

## Data Availability

The datasets used and/or analyzed during the current study are available from the corresponding author on reasonable request.

## Abbreviations

ESCC: Esophageal squamous cell carcinoma
EMT: Epithelial-mesenchyme transition
EZH2: Enhancer of zeste homolog 2
E-cadherin: Epithelial cadherin
Zeb2: Zinc finger E-box binding homeobox 2
Vim: Vimentin
GAPDH: Glyceraldehyde-3-phosphate dehydrogenase
