# Author Correction: EZH2 regulates oncomiR-200c and EMT markers in esophageal squamous cell carcinomas

**DOI:** 10.1038/s41598-025-07153-9

**Published:** 2025-06-24

**Authors:** Fatemeh Nourmohammadi, Mohammad Mahdi Forghanifard, Mohammad Reza Abbaszadegan, Vajiheh Zarrinpour

**Affiliations:** 1 Department of Biology, Damghan Branch, Islamic Azad University, Damghan, Iran; 2 Medical Genetics Research Center, Mashhad University of Medical Sciences, Mashhad, Iran

Correction to: *Scientific Reports* 10.1038/s41598-022-23253-2, published online 31 October 2022

The original version of this Article contained an error in Figure 1 and Figure 6c, where the GFP control and certain ICC images were mislabelled due to an unintentional error by the photography operator during image preparation.

The original Figure 1 and Figure 6 and accompanying legend appear below.

**Fig. 1:**
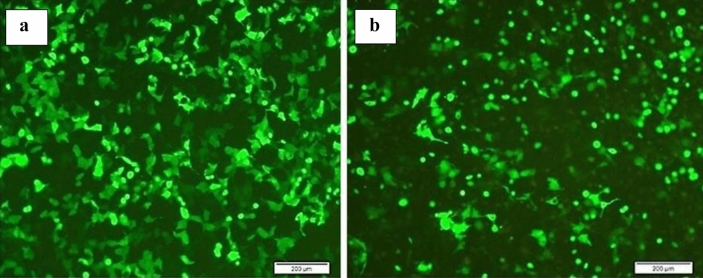
Invert and fluorescent microscope images of GFP transfection efficiency of GFP gene expression in (**a**) YM1 and (**b**) KYSE-30 cells. Fluorescent microscopy images of GFP control cells 24 h after transfection.

**Fig. 6:**
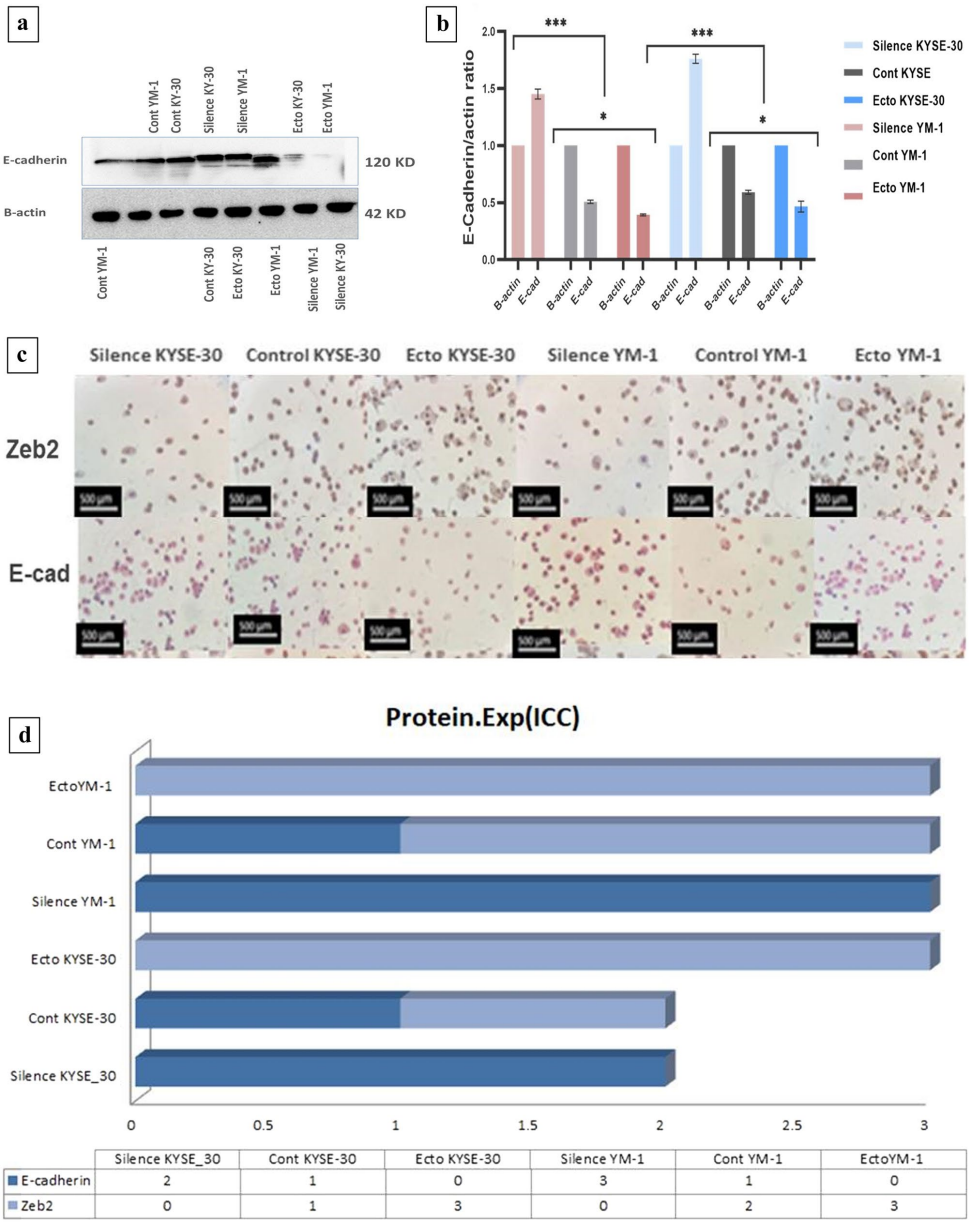
(**a**) WB Image analysis with the G-Box gel documentation system. (**b**) WB analysis confirmed high level of E-cad protein experssion in EZH2-ectopic cells. B-actin antibodies was used as a control. (**c**) Positive staining corresponds to Iimmunocytochemistry staining of E-cad and Zeb2 protein (dark brown is strong positive). (**d**) patologist score to E-cad and Zeb2 protein expersion via Immunocytochemistry assay (3 is srongly positive) (Supplementary Information).

The original Article has been corrected.

