# Supplementary material for: EZH2 regulates oncomiR-200c and EMT markers in esophageal squamous cell carcinomas
Source: Sci Rep. 2022 Oct 31;12:18290. doi: 10.1038/s41598-022-23253-2 (PMC9622866; doi:10.1038/s41598-022-23253-2)
Supplement: Supplementary file 1 — Supplementary Information. [file 41598_2022_23253_MOESM1_ESM.docx]

**Cont Ym-1**

**Cont KY-30**

**Silence KY-30**

**Silence YM-1**

**Ecto YM-1**

**Ecto KY-30**


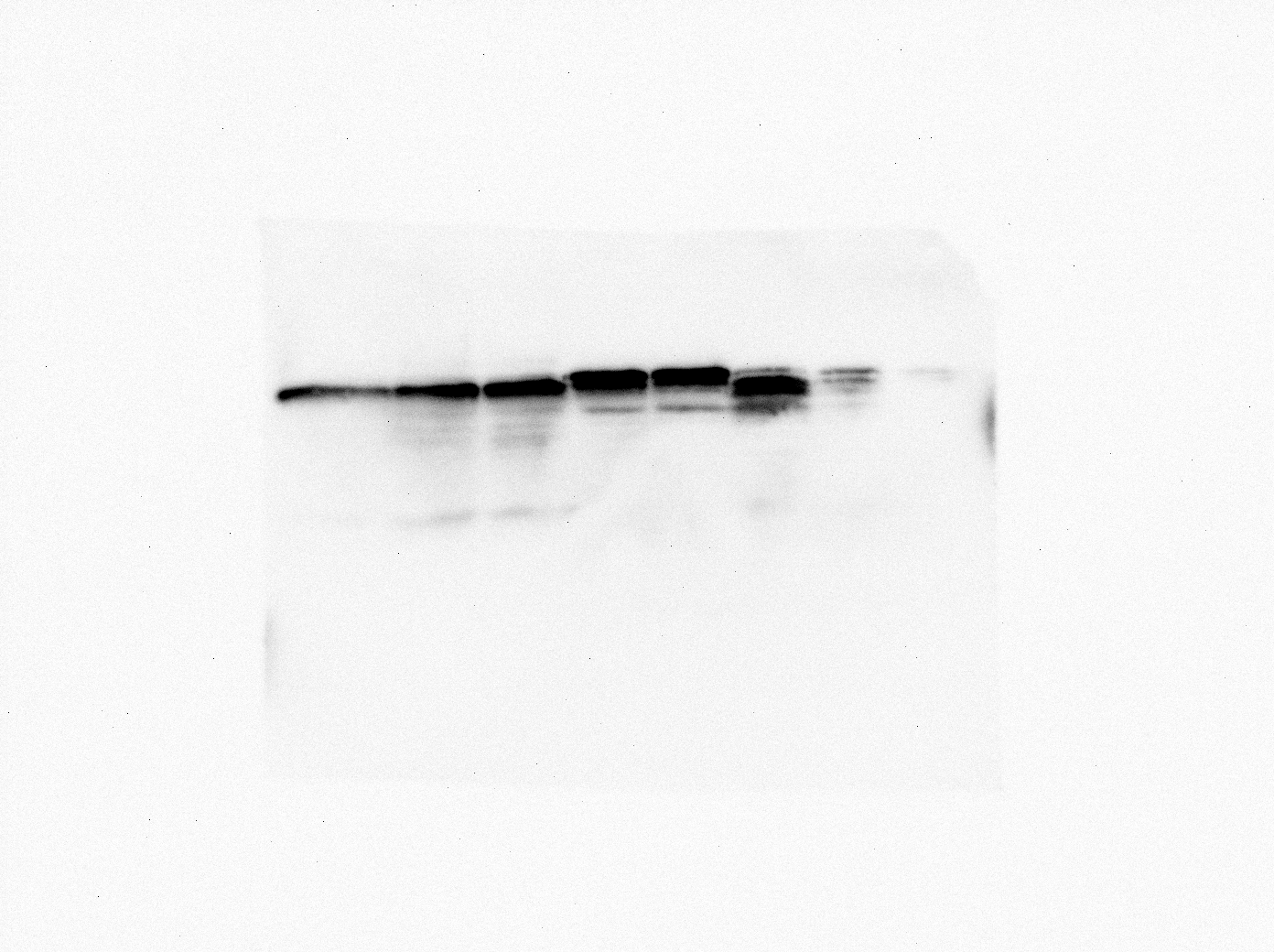


**
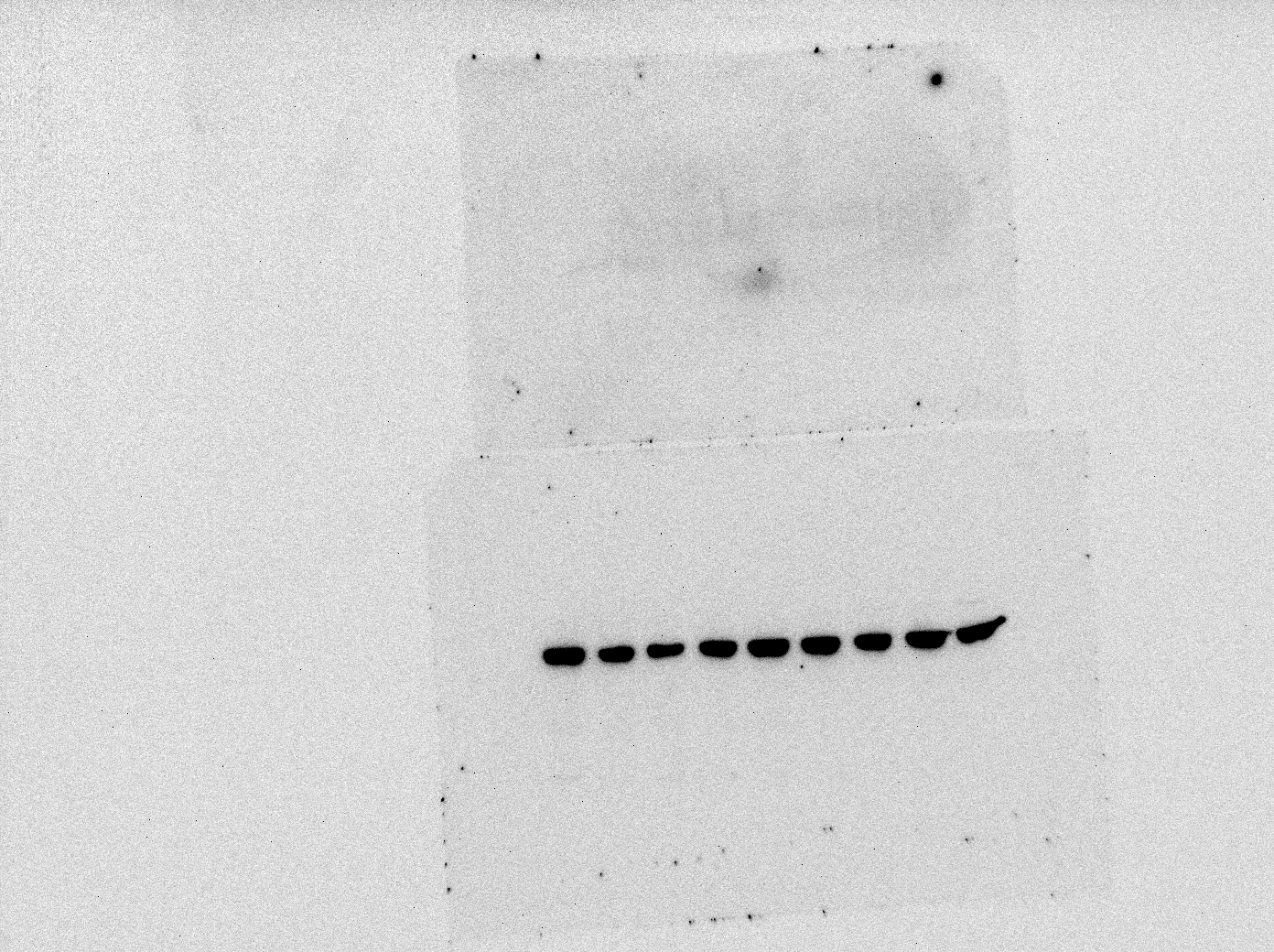
E-cadherin**

**Cont Ym-1**

**Cont KY-30**

**Ecto KY-30**

**Ecto YM-1**

**Silence KY-30**

**Silence YM-1**

**B-actin**
